# mRNA expression analysis of the hippocampus in a vervet monkey model of fetal alcohol spectrum disorder

**DOI:** 10.1186/s11689-022-09427-z

**Published:** 2022-03-19

**Authors:** Rob F. Gillis, Roberta M. Palmour

**Affiliations:** 1 Department of Human Genetics, McGill University, Montreal, Canada; 2 Department of Psychiatry, McGill University, Montreal, Canada; 3 Behavioural Science Foundation, St. Kitts, Federation of St Kitts and Nevis, Montreal, Canada

**Keywords:** Fetal alcohol spectrum disorders, Gene expression analysis, Neurodevelopment, Non-human primate model

## Abstract

**Background:**

Fetal alcohol spectrum disorders (FASD) are common, yet preventable developmental disorders that stem from prenatal exposure to alcohol. This exposure leads to a wide array of behavioural and physical problems with a complex and poorly defined biological basis.

Molecular investigations to date predominantly use rodent animal models, but because of genetic, developmental and social behavioral similarity, primate models are more relevant. We previously reported reduced cortical and hippocampal neuron levels in an Old World monkey (*Chlorocebus sabaeus*) model with ethanol exposure targeted to the period of rapid synaptogenesis and report here an initial molecular study of this model. The goal of this study was to evaluate mRNA expression of the hippocampus at two different behavioural stages (5 months, 2 years) corresponding to human infancy and early childhood.

**Methods:**

Offspring of alcohol-preferring or control dams drank a maximum of 3.5 g ethanol per kg body weight or calorically matched sucrose solution 4 days per week during the last 2 months of gestation. Total mRNA expression was measured with the Affymetrix GeneChip Rhesus Macaque Genome Array in a 2 × 2 study design that interrogated two independent variables, age at sacrifice, and alcohol consumption during gestation.

**Results and discussion:**

Statistical analysis identified a preferential downregulation of expression when interrogating the factor ‘alcohol’ with a balanced effect of upregulation vs. downregulation for the independent variable ‘age’. Functional exploration of both independent variables shows that the alcohol consumption factor generates broad functional annotation clusters that likely implicate a role for epigenetics in the observed differential expression, while the variable age reliably produced functional annotation clusters predominantly related to development. Furthermore, our data reveals a novel connection between *EFNB1* and the FASDs; this is highly plausible both due to the role of EFNB1 in neuronal development as well as its central role in craniofrontal nasal syndrome (CFNS). Fold changes for key genes were subsequently confirmed via *q*RT-PCR.

**Conclusion:**

Prenatal alcohol exposure leads to global downregulation in mRNA expression. The cellular interference model of *EFNB1* provides a potential clue regarding how genetically susceptible individuals may develop the phenotypic triad generally associated with classic fetal alcohol syndrome.

**Supplementary Information:**

The online version contains supplementary material available at 10.1186/s11689-022-09427-z.

## Background

The effect of alcohol consumption in utero was first documented in the modern era between 1968 and 1973 based on a cohort of children that all shared a specific pattern of cranio-facial dysmorphology, central nervous system defects and growth deficiency which led to the initial phenotypic characterization of fetal alcohol syndrome (FAS) [1, 2]. As time progressed, it became evident that the criteria for FAS were too restrictive and did not capture the full range of effects that pre-natal ethanol exposure might induce in a developing child. This led the nosology through a series of updates until the term fetal alcohol spectrum disorders (FASD) was created to satisfy the full range of damaging effects that alcohol may have on a developing embryo [3]. The major risk factors for developing clinical FASD appear to be a combination of the gestational timing of ethanol exposure, degree of ethanol exposure [4], and a genetic predisposition to the effects of prenatal alcohol exposure (PAE) [5–8]. The diverse nature and stochasticity of these risk factors coupled with the lack of full compliance limits the ability of public health agencies to rely exclusively on information campaigns to prevent this disorder. As a result, attention and focus must be placed on understanding its development from a molecular perspective to generate an alternate route of intervention.

A promising strategy to identify the impact of PAE on neurodevelopment involves the use of gene expression analysis in model organisms. Several studies utilizing this strategy have implicated a wide variety of genes and a diverse array of cellular processes without the clear emergence of an underlying consistency [9–21]. The wide range of cellular processes implicated in these various studies likely stems, at least in part, from the very high number of study design combinations (different organisms, amount, pattern and gestational timing of exposure, tissues interrogated, and acute vs temporally delayed measurement). These combinations in study designs may yield valuable yet distinct results that are difficult to assimilate into a coherent picture of such a heterogeneous disorder. This diversity and lack of a clear molecular pathway may also not be surprising given that ethanol appears to have relatively non-specific, sometimes transient, low affinity interactions with its targets as opposed to compounds with a specific binding domain represented in a single peptide family [22]. The level of clinical heterogeneity observed in patients with FASD would be expected given range of gene expression effects in animal models of FASD, yet amongst the clinical heterogeneity are several re-emerging phenotypes that enabled the initial characterization of FAS and FASD.

The hippocampus is a particularly relevant structure to examine due to the well documented effects that ethanol exerts in this vulnerable region both pre- and postnatally. Early studies showed a reduction in the number of dendrite arborizations in the hippocampus after long-term alcohol abuse [23]. Others have shown that there is a reduction in the number of glial cells in the hippocampus of chronic alcohol abusers [24]. The adolescent brain shows increased hippocampal neurotoxicity related to alcohol consumption [25] and it has also been shown that hippocampal volume is decreased in adolescents with chronic exposure to alcohol [26–28]. There have been several reports of increased cell death and decreased volume in the hippocampus due to PAE in rodents [29, 30] and guinea pigs [31]. In addition, our group has previously reported significant numerical reductions in the principal hippocampal neurons of *C. sabaeus* offspring with moderate levels of PAE during the period of rapid synaptogenesis [32]. In these animals, the neuronal deficits are present neonatally, persist through infancy (5 months) and increase in juvenile (2 years) stages.

There are several advantages to using this same non-human primate as the model organism in which to study hippocampal mRNA expression changes. First, it allows a simulation of the broader effects observed in FASD in a model organism with developmental trajectory that closely parallels that of humans but is temporally faster. In addition, a substantial proportion (but not all) of the St. Kitts *C*. s*abaeus* monkeys voluntarily drink moderate or even large quantities of beverage in the absence of dietary restrictions or behavioral training, with population level variance ranging from pathological to abstinent similar to that observed in human studies [33].

With respect to the phenotype of animals included in this study, our goal was explicitly to model the changes that would occur during the period of active synaptogenesis. Accordingly, these PAE offspring have neither facial dysmorphologies nor growth retardation. Behaviors characteristic of social dysfunction can be identified as early as 5 months of age, but cognitive impairment (as measured by object retrieval testing) is not robustly identified until 3 or 4 years of age. A more sensitive early measure is neuronal number in PAE offspring as compared to controls. All PAE animals used in this study had alcohol exposure (both absolute levels and developmental periods) shown in our anatomical studies to produce a 40% reduction in frontal cortical neurons [34] and nearly 60% reduction in CA1 hippocampal neurons at 2 years of age [32]. These characteristics allow us to unite the features that are more applicable to humans regarding this disorder yet still enable the controlled manipulation of key experimental variables. Using this primate model, we interrogated the mRNA expression of the entire hippocampus in both PAE and control individuals using GeneChip microarray technology. These characteristics allow us to unite the features that are more applicable to humans regarding this disorder yet still enable the controlled manipulation of key experimental variables. Using this primate model, we interrogated the mRNA expression of the entire hippocampus in both PAE and control individuals using genechip microarray technology.

## Methods

### Animal care

Male vervet monkeys of two age categories [5 months infants (mean 5.6 months ± 0.87 months); 2-year juveniles (mean 26.4 months ± 2.68 months)] and two conditions (FASD, control) were selected for this study (GSE173516). Male vervets were examined exclusively in order to eliminate any biological variance related to sex [33, 35, 36]. All animals were captive-bred under the care of the Behavioural Science Foundation (St. Kitts, Eastern Caribbean) and housed within outdoor social enclosures in a setting that resembles their natural environment with regular foraging opportunities. These subjects were fed with High-protein Primate Chow (Harlan, USA) and local produce with unlimited access to clean drinking water. To avoid the stress of forced alcohol administration, only alcohol preferring dams were used in this study.

All procedures in this study were reviewed and approved by the Animal Care Committee of McGill University (Montreal, Canada, protocol #4627) and assented to by the Animal Care Committee of Behavioural Science Foundation (BSF 1103, 1301), both acting under the auspices of the Canadian Council on Animal Care. Behavioural Science Foundation conducts research using standard operating procedures for all in vivo procedures described in this study.

### Alcohol exposure

As noted in the abstract, many St. Kitt’s vervets will voluntarily drink intoxicating amounts of beverage alcohol [36]. However, this propensity is not universal, and population studies indicate that the prevalence of alcohol preference in *C. sabaeus* closely mimics that seen in human populations [34]. Specifically, about 25% of the population will voluntarily drink to intoxication and approximately 5% drink abusively. Prior to the present study, adult females that would drink at least 2 g of beverage ethanol in a 4-h scheduled access period were identified, using a two-choice bottle method previously described [37], all specific data related to age and exposure is available in the online data [https://doi.org/10.5061/dryad.g1jwstqqz]. Social groups comprising 5–6 alcohol preferring dams, as defined above, were housed with a single alcohol avoiding male. Once group stability had been established, groups were observed for evidence of menstrual cycling and reproductive behaviour. At approximately 1 month after breeding was observed, females were examined, and the uterus was measured and palpated for evidence of pregnancy. Animals that were pregnant were shave-marked for rapid identification, and gestational stage was followed by semi-weekly physical examinations. Alcohol was presented to selected dames 56 to 77 (mean 67 ± 8) days prior to the birth of full-term infants. During the alcohol administration period, the door between the main cage and the drinking compartment was opened, and the dam either walked directly into the drinking compartment or was encouraged to do so by presentation of a piece of banana or other fruit. Pregnant dams were then offered either an ethanol solution (PAE condition: 8% w/v ethanol in tap water) or an equal volume of isocaloric sucrose with no ethanol (control condition). The same alcohol-preferring females might be offered ethanol during one breeding cycle, and sucrose during the next, or vice versa. Tap water was freely available to all animals at all times. Volumes of the ethanol solution were varied so that each alcohol exposed mother would be allowed to drink no more than 3.5 g ethanol per kg body weight in a single session. At the end of the 4-h exposure period, all animals were returned to the social group. A small amount of blood was collected from both alcohol-drinking and control dams trained to present a leg for unanesthetized blood collection every 2 weeks for the measurement of blood alcohol concentration.

### Tissue collection

Age-matched cases and controls were scheduled for sequential sacrifice and moved from social groups to individual cages several days prior to sacrifice in order to minimize environmental sources of variance. Animals were sacrificed between 10 am and noon to minimize variance related to circadian oscillation. Sacrifice occurred under ketamine anesthesia, using an American Veterinary Medical Association-approved pentobarbital solution to minimize distress [38]. Furthermore, animals from within each group were randomly assigned a sacrifice order with an alternation between cases and controls. Brains were perfused with ice-cold RNAse-free phosphate-buffered saline, rapidly removed, and dissected with sterile instruments rinsed in sterile RNAse-free phosphate buffered saline. The left hippocampus was removed subsequent to a saggital cut through the brain separating the left and right hemispheres and transferred to a sterile vial containing 1 ml Guanidine isothiocyanate/phenol solution (Qiazol–Qiagen, Germany) for each 100 mg of tissue. The tissue was immediately homogenized using a portable homogenizer (Tissue Master 125–Omni, USA). Aliquots (1 ml) were transferred to sterile cryovials and frozen at – 40 °C prior to dry-ice shipment to Montreal for further processing and analysis.

### RNA preparation

Total RNA was extracted from the frozen homogenate using the Qiagen miRNeasy kit (Qiagen, Germany), under RNAse-free conditions, following the manufacturer’s protocol. These samples were then transferred to the McGill University and Genome Quebec Innovation Center (Montreal, Canada) for quality analysis and microarray hybridization. Purified total RNA (miRNA and mRNA) was analyzed for concentration using a NanoDrop ND-1000 spectrophotometer (Thermo Scientific, USA) and quality using the Bioanalyzer 2100 (Agilent, USA). The RNA integrity number (RIN) number is influenced by low oligomer length miRNAs and therefore the quality was assessed by combining high RIN numbers with a steady baseline and distinct spikes for each RNA species (miRNA, 18s,28s) while examining the electropheretograms generated by the 2100 system. Furthermore, complementary RNA (cRNA) quality was also assessed using the Agilent Bioanalyzer and all cRNA samples used in the final study passed this stage of quality control as well. A total of 32 male vervet RNA samples were screened with 24 selected for final array hybridization based on preferred sample quality. These 24 samples were equally divided between each of four groups: 6 (5-month FASD), 6 (5-month control), 6 (2-year FASD), and 6 (2-year control).

### Array hybridization, quality control, analysis

Samples were hybridized to Affymetrix GeneChip Rhesus Macaque Genome Arrays at the McGill University-Genome Quebec Innovation Center (MUGQIC). The RNA samples were all loaded onto the arrays on the same day, in a random manner with respect to biological grouping. The array data were then examined for evidence of RNA degradation using the AffyRNAdeg function in the Bioconductor package [39] developed for the open-sourced software environment R (www.R-project.org) (Supplemental Figure 1). The raw data was normalized using the robust multi-array average (RMA) normalization method [40]. These data were also explored further with the FlexArray software package [41] developed at the MUGQIC. All arrays passed initial quality control however, as evidenced by 3D principal components analysis (Supplemental Figures 2, 3), two arrays displayed strong outlier profiles (one 5-month FASD and one 2-year FASD) and were eliminated from further analysis. The final sample thus had an unbalanced 2 × 2 design with 22 arrays.

### Probe filtering

Probe sets that could not be annotated were filtered out using combined information from the available Affymetrix annotation and the private annotation developed by the Norgren lab at the University of Idaho. This reduced the number of probe sets from 52,866 to 24,402. The remaining probe sets were further reduced using the MAS 5.0 Absolute Detection function in the Affymetrix array suite for R [42], such that only probe sets that showed significant expression levels (*p* value < 0.05) in at least 6 arrays were included for further analysis. This reduced the number of probe sets from 24,402 to 17,652. Following this, genes for which multiple probe sets were present were reduced to minimize the number of tests performed by selecting the probe set with the highest mean expression level across all arrays. This method was chosen given the nature of cross species hybridization as well as the unreliable nature of many of the additional probe sets on the array [43]. This reduced the total number of probe sets corresponding to unique genes to 11,512. These 11,512 annotated, expressed and unique gene entries were carried forward for statistical analysis.

### Statistical analysis

Consistent with the research design, these retained datasets were analyzed using a two-way analysis of variance (ANOVA), with two main factors (alcohol, age) each with two levels (5 month/2 years, FASD/control). The analysis strategy was carefully conceived a priori, and the gene annotations were not consulted until we were satisfied these data had been properly analyzed. This is a subtle distinction that ensured we would not analyze these data via multiple avenues before selecting the results which produced the most pleasing results. Constrained exploration of the array was performed by creating a narrowed subset of genes of interest a priori (the candidate pool) which was assigned *q*-values separately from the general pool in order to minimize the difficulties associated with multiple comparisons [44]. This method of constrained exploration was only utilized for the independent variable of interest (alcohol) to increase our ability to detect important and relevant signals. The *p* values were converted to *q* values as a means to represent the false discovery rate given the number of tests performed within this study [45]. The *q* value is a useful algorithm to correct for multiple testing given that with traditional FDR cannot be defined when there are no positive results. A recent update to this algorithm using an additional informative variable approach is available for processes such eQTL mapping or RNA-seq data where an additional variable such as read depth can enhance the overall amount of information available to calculate these *q* values [46]. The remaining genes (the general pool) were still examined to avoid ignoring potentially important surprise findings; however, their *q* values [45] were calculated independently from those of the candidate pool. The candidate pool was chosen a priori after reviewing gene ontology (GO) terms with high probability of involvement in the FASD phenotype (retinoic acid signalling, WNT signalling, CNS development, synaptic transmission, maturation, establishment, axon guidance, netrin receptor activity, cholesterol homeostasis, activator of MAPK activity, caspase activity), and included genes that were identified as differentially expressed in previous studies of FASD that had been performed prior to 2013.

### qRT-PCR

Five differentially expressed genes were selected from the two independent variables; [*EFNB1, GGCT* (alcohol) and *GBPB1L1, RHPLN1, SOX4* (age)] as a means to validate the array data. Primers for amplification were developed using genomic sequence data from *Chlorocebus sabaeus,* the model organism used within this study. The sequences used for amplification are listed in Supplemental Table 1. The mean values of *ACTB* were used to generate delta Ct values. RNA quality and cDNA quality were analyzed using the Bioanalzyer 2100 (Agilent, USA) at the Institut de Recherche en Cancerologie et Immunologie (IRIC, Montreal, Que). The *q*RT-PCR reactions were executed on the Illumina HiSeq 2000 using a TaqMan assay design with a no-reverse transcriptase and H_2_O as negative controls. The 2^–∆∆Ct^ method was used to calculate fold changes in FASD animals relative to controls for *EFNB1* and *GGCT* and 5 months relative to 2 years for *SOX4*, *GBPB1L1* and *RHPN2*.

### Functional annotation

Functional annotation was performed using the database for annotation, visualization and discovery (DAVID, v 6.8) by creating a custom background of total expressed genes from our tissue and a differentially expressed gene list with a *p* value threshold of 0.016 corresponding to a total of 297 genes from both the candidate and general pools. This gene list was created using the lowest raw *p* values from both candidate and general pools to avoid introducing a tautology to the gene list based on our a priori screening. The Entrez gene ID was used for both the differentially expressed gene list as well as the custom background. Functional annotation clustering was performed using the gene ontology (GO) term FAT selected to eliminate broad matches. The process was repeated using age as an independent variable using the same number of differentially expressed genes (297) to compare the output between our two variables.

## Results

The study design enabled the exploration of two independent variables (age, alcohol) as well as their interaction. The overall data pattern of the arrays revealed very high pairwise correlation between arrays with a minimum of 0.94 and a mean of 0.97 with low variance and low overall fold changes for both of our experimental factors (age, alcohol) indicating that we managed to control many sources of external variance. A table of mean expression and mean variance is available as Supplemental Table 2. These fold changes are low in comparison with microarray studies where the phenotype (e.g., cancer) is more severe or in cases where it is possible to compare diseased tissue to normal tissue from the same individual. Despite the low distinction between groups and the strong correlation between the arrays, it is clear from a histogram of *p* values (Supplemental Figures 5 and 6) that each of our independent variables produced a notable within group effect on gene expression that differs from the null hypothesis.

### Independent variable: alcohol

**Fig. 1:**
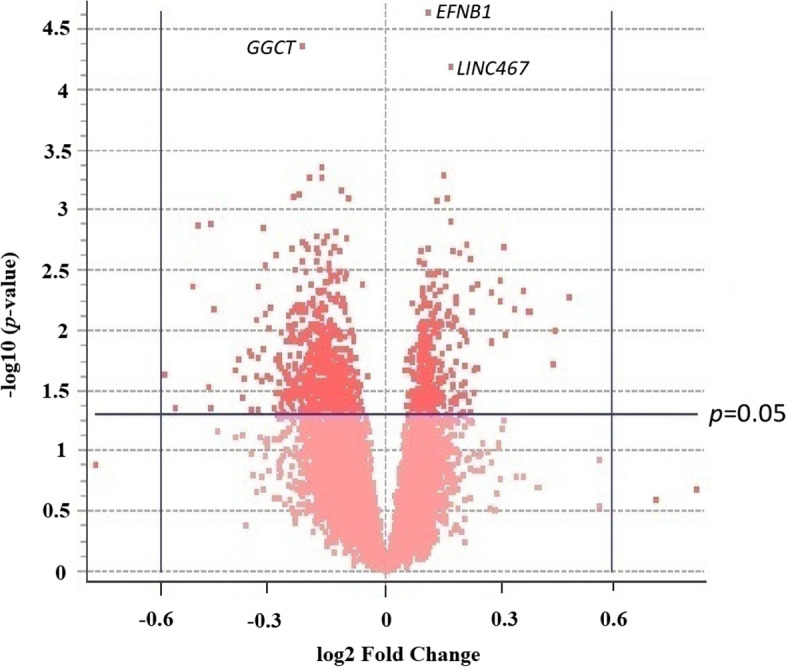
Volcano plot displaying the relationship between fold change (log2) and p-value (-log10) for all probe sets from both the a priori candidate pool and the general pools using Alcohol as an independent variable between pregnant vervet dams which voluntarily consumed alcohol between e100-165 and their sucrose matched controls. There was an overabundance of down regulated genes vs upregulated genes exploring the effect of alcohol. *EFNB1* had the lowest p-value on the array and is identified at the top of the figure

Exploring the effect of alcohol as an independent variable, there were 938 (116 from candidate, 822 general pool) total genes with a *p* value < 0.05, as compared to the 575 genes with a *p* value < 0.05 expected under the null hypothesis (Supplemental Figure 5). The *p* value for this departure in expected vs observed number of genes in this range is 3.6 × 10^−54^. This represents an over representation of 363 genes that have *p* values in this range. Specific exclusion of false positives is rendered difficult given the number of tests performed within an array where over 11,000 genes will be interrogated. This challenge was anticipated and led us to create a candidate gene list a priori for alcohol as an independent variable which pared the number of tests within that cohort of genes. Therefore, *q* values within this a priori candidate gene list were calculated independently of the remaining genes that were listed within a general pool. Of the 938 genes that achieved *p* < 0.05 for alcohol, 618 were down regulated and 320 were upregulated (Fig. 1) which is inconsistent with a 50/50 distribution (*p* value = 2.24 × 10^−22^). This is in contrast with the balanced number of differentially expressed upregulated and downregulated genes influenced by age indicating that excess downregulation was specific to alcohol. Overall, the data pattern supported a role for the probe set corresponding to *EFNB1* within the candidate pool having an outsized effect relative to the background, with a *q* value of 0.0297 (*p* value 2.27 × 10^−5^). The gene with the next lowest *p* value of 4.3 × 10^−5^ corresponds to *GGCT* (*q* value of 0.26) which had been initially identified in the general pool. The third lowest was *LINC476*, a locus that does not code protein.

**Fig. 2:**
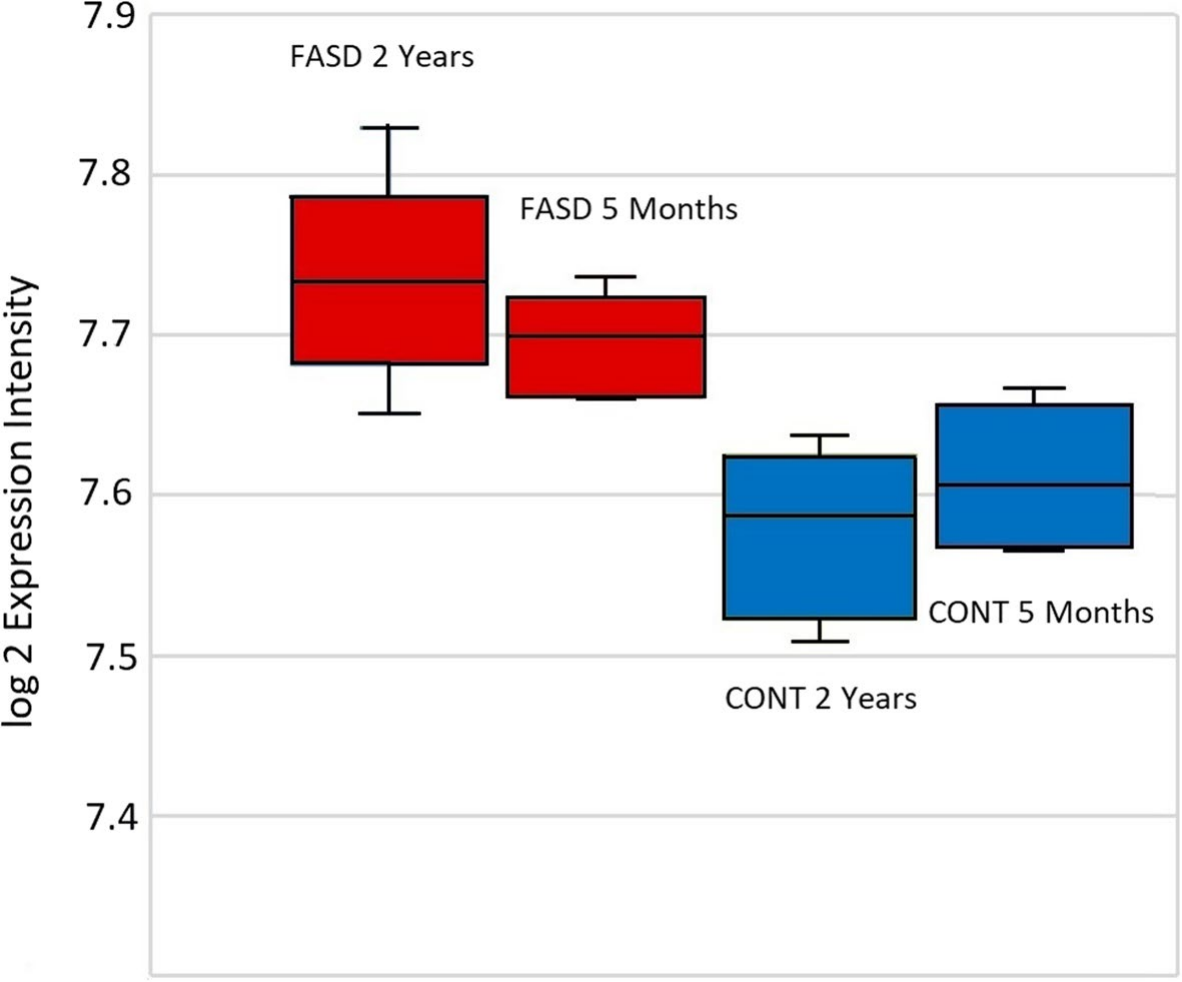
This figure shows the box plots of log2 expression intensity for* EFNB1* across the four study groups within this data set showing the mean log2 expression of *EFNB1* is higher in alcohol exposed monkeys relative to their sucrose matched controls. The interaction effect for *ENFB1* was not significant

The expression profile was higher for *EFNB1* in both 5-month and 2-year-old monkeys exposed to alcohol in utero as seen in Fig. 2. The *p* value for the interaction effect between age and alcohol was low (0.09) but not significant indicating that the upregulation of *EFNB1* remained relatively constant as the monkeys continued through this period of development.

Pathway exploration and functional annotation was performed on the 297 genes with the lowest *p* value on the array using the DAVID functional annotation tool. The full table of results can be found in the supplemental data files that are accessible online however the two annotation clusters with the highest enrichment scores are presented in Table 1.

**Table 1:** Top two functional annotation clusters generated from a list of 297 genes using alcohol as a independent variable with a p-value cut-off below 0.016

**Annotation cluster 1**	***p*** **value**	**FDR**
MIS complex (methyltransferase)	.00368	0.71
mRNA editing complex	.00368	0.71
Methyltransferase complex	.00397	0.71
mRNA methylation	.0128	1
mRNA modification	.037	1
RNA methylation	.0448	1
Nuclear speck	.148	1
RNA modification	.265	1
**Annotation cluster 2**	***p*** **value**	**FDR**
Chromatin organization	.013	1
Chromosome organization	.0138	1
Covalent chromatin modification	.0329	1
Histone modification	.0387	1
Chromatin regulator	.0281	1

### Independent variable: age

**Fig. 3:**
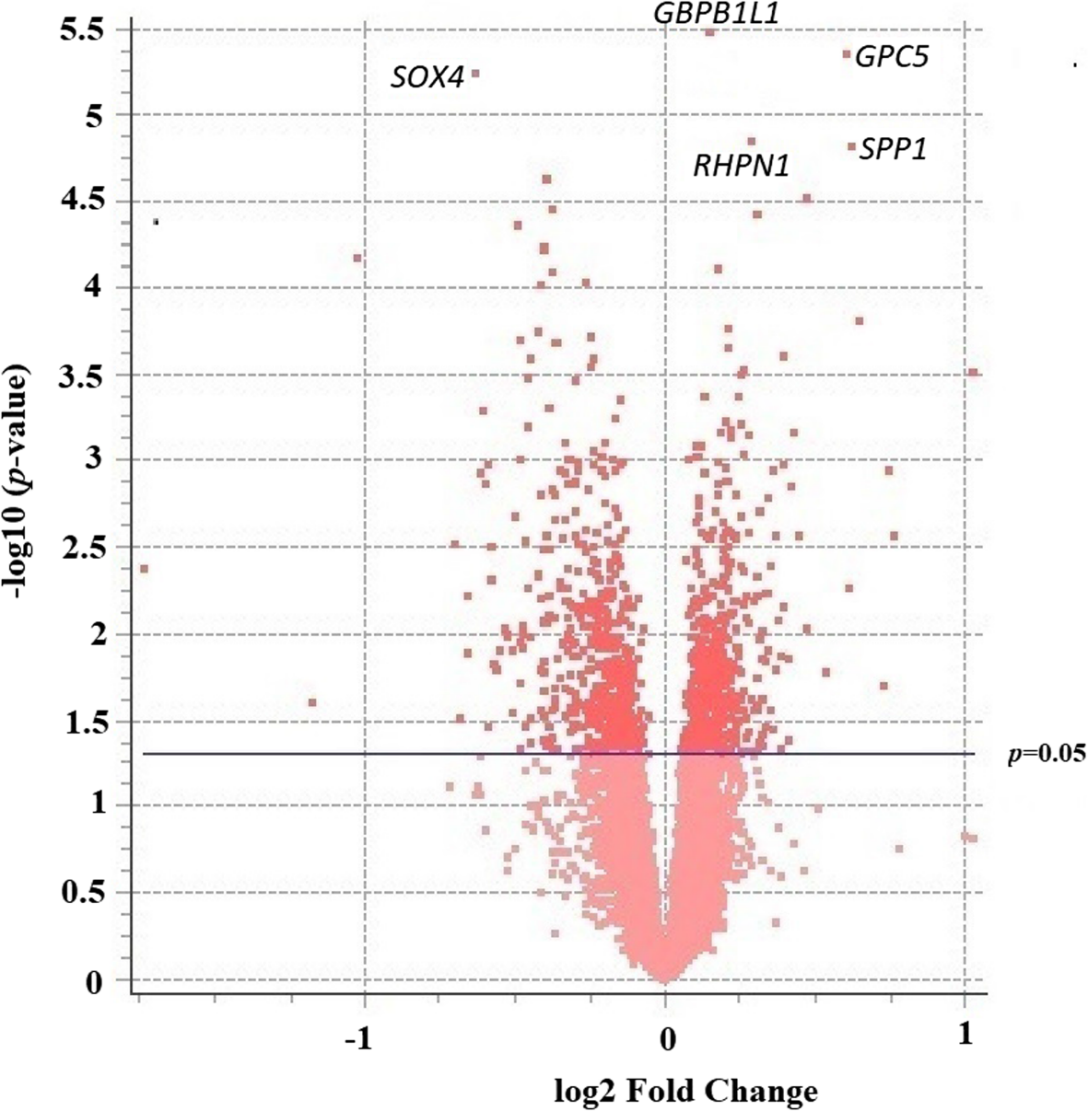
Volcano plot displaying relationship of p-values with fold changes using Age as an independent variable. Each dot represents a gene which has been plotted against the -log 10 of its p-value (vertical axis) and log 2 fold change (horizontal axis). There is a relative balance between up-regulated genes and down regulated genes using Age as an independent variable

Examining the effect of age on gene expression, we generally observed much lower *q* values in comparison to the alcohol phenotype, and a greater number of genes which display a *p* value less than 0.05. The number of genes below this threshold totalled 1055 (*H*_0 =_ 575), revealing an additional 480 genes than would be expected under the null hypothesis. In contrast with alcohol as an independent variable, there was a relative balance between upregulated and down regulated genes with 534 downregulated and 521 upregulated. The volcano plot below displays the relationship between fold change and *p* value for all mRNAs interrogated in this study using alcohol as an independent variable (Fig. 3).

We also performed functional annotation clustering by creating a gene list of 297 genes with the lowest *p* values using age as an independent variable (Supplemental data files). In general, the *q* values for these genes were lower ranging from 0.0189 to 0.3 as opposed to 0.297–0.49 in the case of alcohol as an independent variable with a far greater number of genes with *q* values below 0.2. Eight of the top 15 annotation clusters were related to development or cell migration with gene ontology (GO) producing lower *p* values than those which were observed using alcohol as an independent variable (Supplemental Table 3).

In addition, the study design allows for the exploration of an interaction effect between age and alcohol which is a powerful method of identifying expression changes related to alcohol exposure that change through time. The interaction between age and alcohol resulted in slightly fewer than 5% of the probe sets with *p* values less than 0.05 with no probe sets having significant *q* values indicating that precise identification of differentially expressed genes which are influenced by alcohol in a manner that changes between 5 months and 2 years is either non-existent or buried within the background noise and unable to be identified without greater statistical power (Supplemental Figure 7). In addition, there were no genes within the interaction term whose *q* values distinguished themselves in a manner that would enable a prudent identification. Therefore, the interaction term was not explored further.

### qRT-PCR

**Fig. 4:**
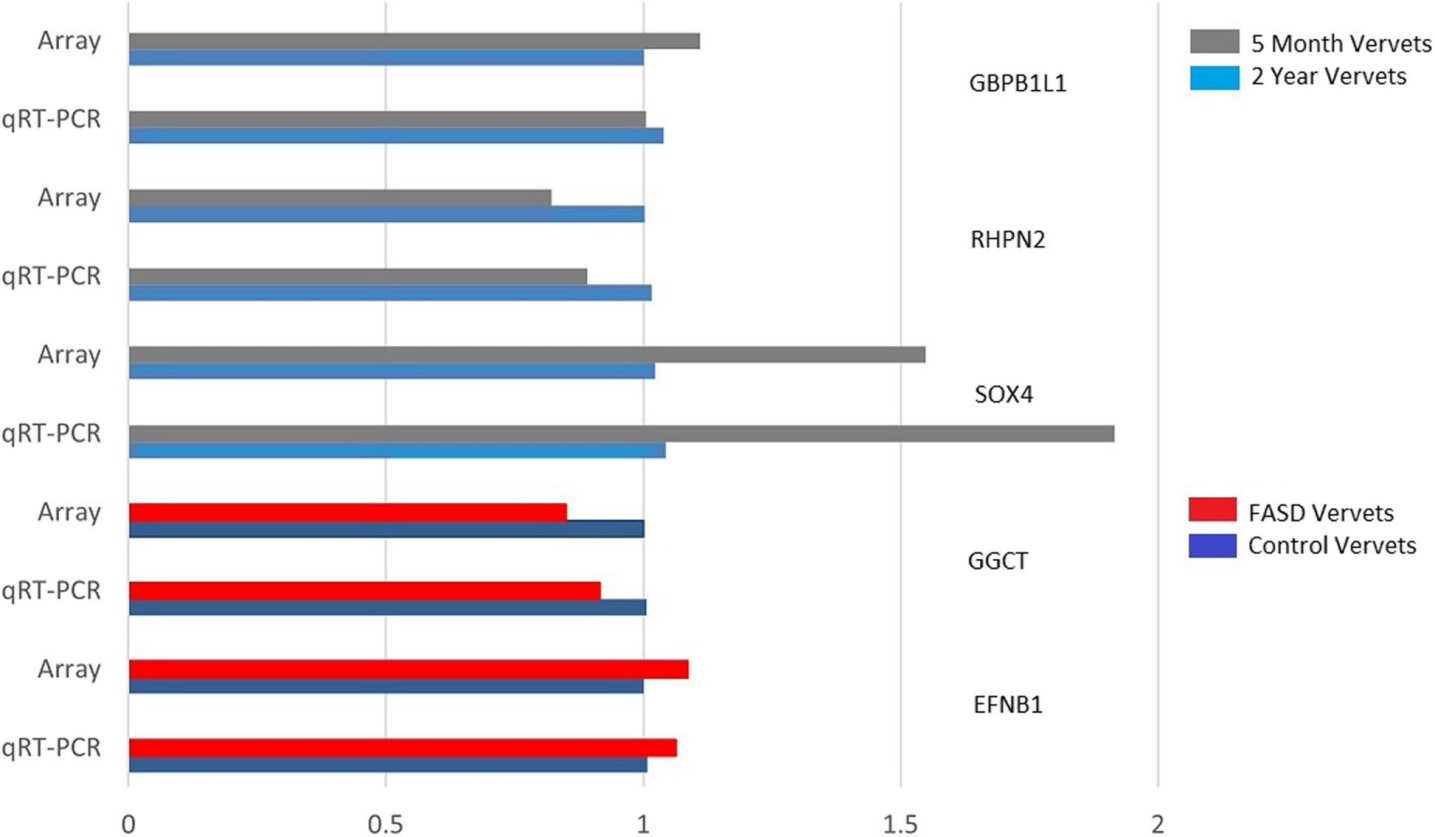
Fold change comparison between data from the GeneChip microarray and qRT-PCR for 5 selected genes. Two genes showing significance for alcohol (EFNB1, GGCT) and three genes for Age (SOX4, RHPN2, GBPB1L1)

Five genes were chosen to validate the gene expression results via their correlation to a *q*RT-PCR platform. The genes were selected based on their low *p*-values using either alcohol or age as an independent variable. The two genes that were selected based on their low *p* values using alcohol as an independent variable were *EFNB1* and *GGCT*. Three genes using age as an independent variable were selected *GBP1L1*, *RHPN2*, and *SOX4*. The 2^–∆∆Ct^ method was used to calculate the fold changes corresponding to these genes which can be contrasted with fold changes calculated from the Rhesus GeneChip microarray in Fig. 4. Fold changes were low overall using both age and alcohol as seen in the volcano plots in Figs. 2 and 3 with *SOX4* showing the greatest fold change among the genes interrogated via qRT-PCR and among the genes with the largest fold change using both age and alcohol on the array overall. *GBPB1L1,* a gene selected due to its low *p* value using age as an independent variable reveals a discrepancy in the fold change and direction between the array and the *q*RT-PCR. It displayed an inverse correlation with the array and furthermore, the -deltaCt vs log2 expression plot (Supplemental Figure 8) implied that the primers bound to the vervet genome at an alternate location. Aside from *GPBP1L1* the fold changes between the array data and the qRT-PCR data were in concordance with each other.

## Discussion

This study design resulted in four primary identifications: (1) the functional annotation of clusters related to epigenetic modification (alcohol) as well as annotations related to development using a gene list generated by using age as an experimental factor; (2) the increased number of genes that were downregulated vs upregulated using alcohol as an independent variable; (3) the lack of a clear interaction effect between alcohol (prenatal vs control) and age at 5 months and 2 years; and (4) the emergence of *EFNB1* as a candidate gene that might be related to some aspects of the phenotypic consistency and genetic vulnerability to PAE in humans. These four identifications will be discussed in further detail below.

We analyzed these data to assess the possibility of a disrupted molecular pathway using alcohol as an independent variable with the functional annotation tool DAVID 6.8. The full list of enriched clusters (found in the online data files) broadly implicates a wide variety of cellular processes with our top two clusters related to epigenetic modifications. These results must be interpreted carefully given the lower false discovery rate (FDR) assigned to them and also because this type of pathway exploration has been performed repeatedly without a clearly replicated pathway that is incontrovertibly linked to PAE [9–18]. Interestingly though, a recent meta-analysis [47] highlighted that despite the dissimilarity across studies, a gene set related to pathways involved in protein synthesis, mRNA splicing, and chromatin organization has emerged and our data, in part, replicates this finding.

Including age as an independent variable within our experimental design provided the opportunity to generate data that would produce results consistent with previously identified GO terms and annotation clusters related to development. In that sense, this experimental factor served as an experimental prediction and the results gave a strong indication of the face validity of the quality of the data. The importance of this predictive nature for one of the independent variables cannot be understated given the potential for gene expression studies, particularly related to PAE, to return unexpected and inconsistent pathway or enrichment results given the number of experimental combinations related to this phenotype.

The second benefit of using age as an independent variable is that it allowed us to explore the interaction between age and alcohol which informs us of whether the gene expression changes are chronic and are altered from the neonate stage through 2 years of simian development or whether they revert to levels observed in control animals as development proceeds through to the juvenile stage. Our results indicated that there is not a discernable interaction effect within our data set. This does not necessarily imply that the interaction effect is non-existent. This finding may stem from a small effect with a lack of statistical power which might have been corrected with larger sample size or the two age points selected for this study had not shown full differentiation in chronic gene expression related to alcohol.

We identified a relative difference in significantly downregulated genes vs upregulated genes in a roughly 2:1 ratio while examining alcohol as an independent variable that was not observed using age as an independent variable. This result is consistent with a recent meta-analysis revealing a systemic down regulation of genes influenced by alcohol [47]. A functional explanation for this observation could be related to an upper level of regulation such as epigenetic modifications or alteration in micro RNA (miRNA) expression levels. Interestingly, early studies into genome wide methylation patterns due to PAE in mice revealed global de-methylation [48]; however, more recently, it has been shown that this pattern is more consistent with demethylation and hyper-methylation depending on the region and the gene in question in both mice [49, 50], as well as in humans [51]. With respect to miRNA, there have been several groups that have already examined the effect of alcohol on miRNA expression in various animal models of FASD (as reviewed in [52]).

We further analyzed a subset of genes using qRT-PCR as a means to develop an independent validation of the results of the microarray analysis. The arrays themselves were highly correlated with each other and displayed very low fold changes, with zero of the genes using alcohol as an independent variable revealing fold changes greater than 2 (log2 = 1) and only 5 of the genes using age as an independent variable revealing fold changes greater than 2 (log2 = 1). Genes that display a fold changes < 2 are typically poor candidates for validation with qRT-PCR [53–56]. Despite this challenge, we replicated the fold changes in four of the five genes interrogated, including *EFNB1*. The fifth gene *GBPB1L1* failed to replicate the fold changes; however, it had an inverse correlation with the expression data from the array and appears to have amplified a different vervet mRNA altogether (Supplemental Figure 8). The low fold changes for all of the genes on the array are consistent with expectations for monkeys that survived and continued through development as opposed to gene expression studies of cancerous tissue or acute challenges with ethanol.

The novel connection between *EFNB1* and FASD provides some potentially exciting insight into the development of FASD as well as the clinical heterogeneity that this disorder presents. *EFNB1* is a receptor tyrosine kinase [57] involved in a large variety of developmental processes such as cell migration, segmentation, and compartment boundary formation, axon guidance, topographic mapping, synaptogenesis, and angiogenesis [58, 59]. The Eph receptors are a family of genes that are critical in neurodevelopment, vascular development, and epithelial development via cell migration, adhesion and pattern formation [60–62]. In addition, *EFNB1* regulates axon guidance [63] and plays a key role in synapse remodelling in the hippocampus [64, 65].

Purely due to its functional role in development, *EFNB1* appears to be an interesting candidate gene for the development of FASD. Perhaps more striking is the resulting clinical presentation that occurs among individuals carrying a mutation in this gene. Loss of function mutations in *EFNB1* lead to craniofronto-nasal syndrome (CFNS) a genetic condition characterized by a wide range of phenotypic effects including frontonasal dysplasia, craniofacial asymmetry, craniosynostosis, bifid nasal tip, grooved nails, wiry hair, and skeletal abnormalities [66–68]. There is a distinct degree of phenotypic overlap between classic FAS and CFNS. Furthermore, there is a wide range of atypical features observed in CFNS that have also been identified among individuals diagnosed with FASD including neurosensory hearing loss and cardiac defects [69–71].

Paradoxically, mutations in this X-linked gene affect females more severely than males, with females prone to the full range of effects while males typically suffer from hypertelorism [66]. The counterintuitive nature of this disorder appears to stem from “cellular interference” [72] that would occur during X-linked inactivation in females where the ephrin B1 signal is skewed across regional developmental boundaries. This skewing across boundaries leads to abnormal cell sorting and ectopic tissue boundaries [73, 74]. Support for this model for CFNS also stems from male children that are mosaic for *EFNB1* mutations that display a more severe phenotype [75]. Interestingly, a genomic duplication of *EFNB1* has been identified in a family segregating for X-linked hypertelorism indicating that development of abnormal craniofacial features is also sensitive to the *expression* level of this gene and not solely dependent on imbalanced signals across tissue boundaries stemming from mutations [76].

The mechanism of cellular interference and the differential expression by which this gene creates such a wide array of overlapping phenotypic features with FASD presents a plausible theoretical model that could reconcile many of the data that has been harvested with respect to FASD. It is well known that ethanol exerts tissue specific effects [77], particularly throughout the brain where it can lead to damaging effects in the hippocampus and frontal cortex [29, 78, 79]. The identification of *EFNB1* presents the possibility that despite the broad mRNA downregulation and epigenetic changes across an array of molecular pathways, the phenotypic craniofacial signatures of classic FAS may emerge periodically from this cellular interference model of *EFNB1* and the tissue specific effects of ethanol due to unbalanced EFNB1 expression levels across tissue boundaries.

The identification of *EFNB1* presents a plausible model for some of the phenotypic features that tend to cluster in patients exposed to alcohol in utero, however caution is warranted until evidence from alternate lines of enquiry can be assimilated into a complete picture of this heterogeneous disorder.

## Conclusions

The vervet monkey provides a powerful non-human primate model for studying the effects of prenatal alcohol exposure in a manner that more accurately reflects the changes that might be observed in humans while simultaneously allowing the generation of a carefully managed case/control environment. This study interrogated male monkeys exclusively to eliminate sex effects, and it is therefore possible that the effect on gene expression in female monkeys would reveal other interesting findings related to pre-natal alcohol exposure. The findings in this study support the frequently observed global downregulation of mRNA due to prenatal alcohol exposure and present a new hypothesis involving *EFNB1* and a cellular interference model that could explain many of the frequently observed phenotypic patterns in humans associated with FASD.

## Supplementary Information

**Additional file 1: Supplemental Figure 1.** RNA degradation plots showing 5’-3’ mean intensity levels for all 24 arrays revealing a similar slope across all samples without any major outliers as a result of 5’-3’ degradation. **Supplemental Figure 2.** Principal component analysis (PCA) of all 24 arrays for the unique expressed probe sets that remained after culling for annotation, multiple probe sets and MAS5 calls. Twenty-two arrays grouped together with FASD5_1 and FASD2_5 showing a distinct expression pattern which skews and distinguishes them from the other arrays due to non-technical variance. These arrays were excluded from further analyses to avoid skewing the group means due to variance unrelated to experimental factors. **Supplemental Figure 3.** 3D PCA plot of expression post normalization via RMA after exclusion of FASD5_1 and FASD2_5 revealing the unsupervised organization of the 22 remaining samples. **Supplemental Figure 4.** Box plots of GeneChip Rhesus Macaque genome array expression data for all 24 arrays after RMA normalization. **Supplemental Figure 5.** Histogram of *p-*value distributions for Alcohol divided into 20 bins with each bin representing 0.05 units. The distribution shows a distinct and sharp increase in the 0-0.05 bin indicating that Alcohol as an experimental factor resulted in a higher number of differentially expressed genes than would have been predicted under the null hypothesis. The black shaded bar represents the most frequent bin within the distribution. **Supplemental Figure 6.** Histogram of *p-*value distributions using Age as a main effect divided into 20 bins with each bin representing 0.05 units of distribution. The distribution shows a distinct and sharp increase in the 0-0.05 bin indicating that Age as an experimental factor resulted in a higher number of differentially expressed genes than would have been predicted under the null hypothesis. The black shaded bar represents the most frequent bin within the distribution. **Supplemental Figure 7.** Histogram of *p-*value distributions for the interaction between Age and Alcohol divided into 20 bins with each bin representing 0.05 units. The distribution shows a flat distribution indicating there is no evidence for a generalized genome wide interaction effect between these two experimental factors. The black shaded bar represents the most frequent within the distribution. **Supplemental Figure 8.** Correlation of log2 expression intensity and -delta Ct values using *ACTB* to normalize expression values. *GBPB1L1* strays from the trend line implying that it may have amplified a target region not represented by the probe set. **Supplemental Table 1.** Mean intensity/expression levels (log2) and mean variance for all 11,512 mRNA for all four groups involved in this study. **Supplemental Table 2.** Primer sequences used for qRT-PCR amplification of selected genes taken from the Rhesus GeneChip array. **Supplementary Table 3.** Functional annotation results using the 297 genes which returned the lowest *p*-values using Age as a main effect. Eight of the 15 top annotation clusters returned results related to development or cell migration with lower overall *p*-values and FDR values when compared to those generated using alcohol as a main effect.

## Data Availability

Raw data files (CEL files) as well as RMA normalized data have been uploaded to the gene expression omnibus website at the National Center for Biotechnology Information using minimum information for a microarray experiment (MIAME) guidelines GSE173516. In addition, all additional data utilized to generate conclusions within this experiment has been uploaded to the dryad database cited at the beginning of the supplemental materials.

## Abbreviations

CFNS: Cranio-frontal nasal syndrome
cRNA: Complementary RNA
DAVID: Database for annotation, visualization, and integrated discovery
FAS: Fetal alcohol syndrome
FASD: Fetal alcohol spectrum disorders
FDR: False discovery rate
GO: Gene ontology
miRNA: Micro RNA
mRNA: Messenger RNA
MUCQIC: McGill University-Genome Quebec Innovation Center
NHP: Non-human primate
PAE: Prenatal alcohol exposure
qRT-PCR: Quantitavie real-time polymerase chain reaction
RNA: Ribonucleic acid
