# Supplementary material for: mRNA expression analysis of the hippocampus in a vervet monkey model of fetal alcohol spectrum disorder
Source: J Neurodev Disord. 2022 Mar 19;14:21. doi: 10.1186/s11689-022-09427-z (PMC8934503; doi:10.1186/s11689-022-09427-z)
Supplement: Supplementary file 1 — Additional file 1: Supplemental Figure 1. RNA degradation plots showing 5’-3’ mean intensity levels for all 24 arrays revealing a similar slope across all samples without any major outliers as a result of 5’-3’ degradation. Supplemental Figure 2. Principal component analysis (PCA) of all 24 arrays for the unique expressed probe sets that remained after culling for annotation, multiple probe sets and MAS5 calls. Twenty-two arrays grouped together with FASD5_1 and FASD2_5 showing a distinct expression pattern which skews and distinguishes them from the other arrays due to non-technical variance. These arrays were excluded from further analyses to avoid skewing the group means due to variance unrelated to experimental factors. Supplemental Figure 3. 3D PCA plot of expression post normalization via RMA after exclusion of FASD5_1 and FASD2_5 revealing the unsupervised organization of the 22 remaining samples. Supplemental Figure 4. Box plots of GeneChip Rhesus Macaque genome array expression data for all 24 arrays after RMA normalization. Supplemental Figure 5. Histogram of p-value distributions for Alcohol divided into 20 bins with each bin representing 0.05 units. The distribution shows a distinct and sharp increase in the 0-0.05 bin indicating that Alcohol as an experimental factor resulted in a higher number of differentially expressed genes than would have been predicted under the null hypothesis. The black shaded bar represents the most frequent bin within the distribution. Supplemental Figure 6. Histogram of p-value distributions using Age as a main effect divided into 20 bins with each bin representing 0.05 units of distribution. The distribution shows a distinct and sharp increase in the 0-0.05 bin indicating that Age as an experimental factor resulted in a higher number of differentially expressed genes than would have been predicted under the null hypothesis. The black shaded bar represents the most frequent bin within the distribution. Supplemental Figure 7. [file 11689_2022_9427_MOESM1_ESM.docx]

Below is a list of supporting figures that are referenced within the main body of the article “mRNA expression analysis of the hippocampus in a vervet monkey model of the hippocampus”. For access to the supplemental data files that are referenced within the main body of the manuscript, as well as full access to all of the data that was used to draw the conclusions within the manuscript, we invite you to visit our data depository at:

https://datadryad.org/stash/share/ej24eEr73GZTbOuWrEcArSvQdnGi48Q2WNym4V6ea7Y


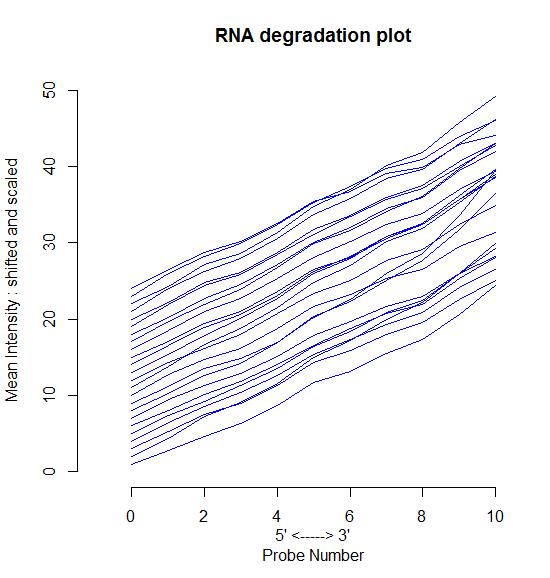


**Supplemental Figure 1:** RNA degradation plots showing 5’-3’ mean intensity levels for all 24 arrays revealing a similar slope across all samples without any major outliers as a result of 5’-3’ degradation


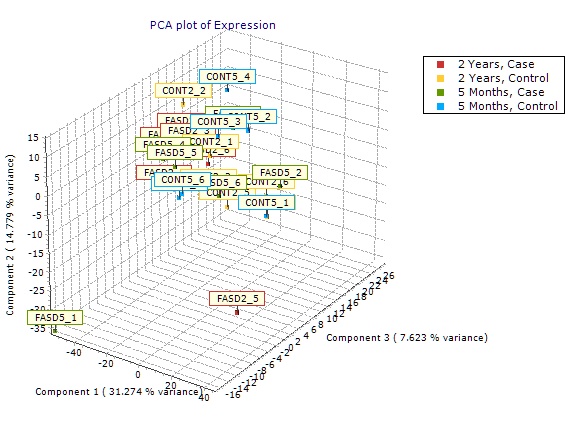


**Supplemental Figure 2:** Principal component analysis (PCA) of all 24 arrays for the unique expressed probe sets that remained after culling for annotation, multiple probe sets and MAS5 calls. Twenty-two arrays grouped together with FASD5_1 and FASD2_5 showing a distinct expression pattern which skews and distinguishes them from the other arrays due to non-technical variance. These arrays were excluded from further analyses to avoid skewing the group means due to variance unrelated to experimental factors.


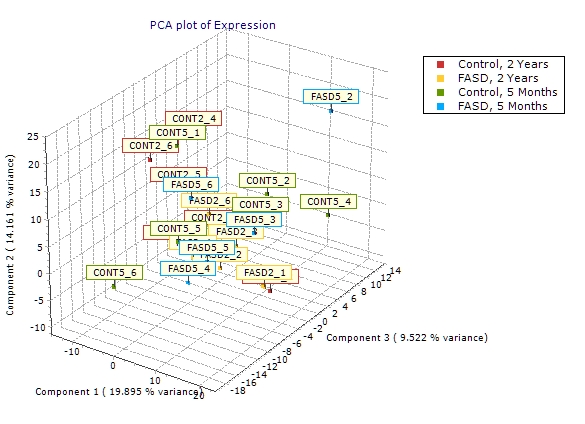


**Supplemental Figure 3:** 3D PCA plot of expression post normalization via RMA after exclusion of FASD5_1 and FASD2_5 revealing the unsupervised organization of the 22 remaining samples.


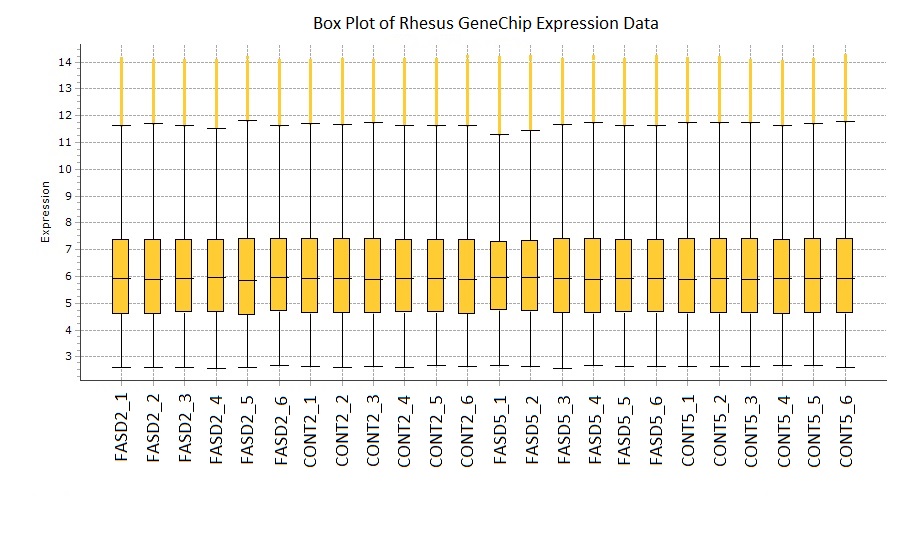


**Supplemental Figure 4:** Box plots of GeneChip Rhesus Macaque genome array expression data for all 24 arrays after RMA normalization


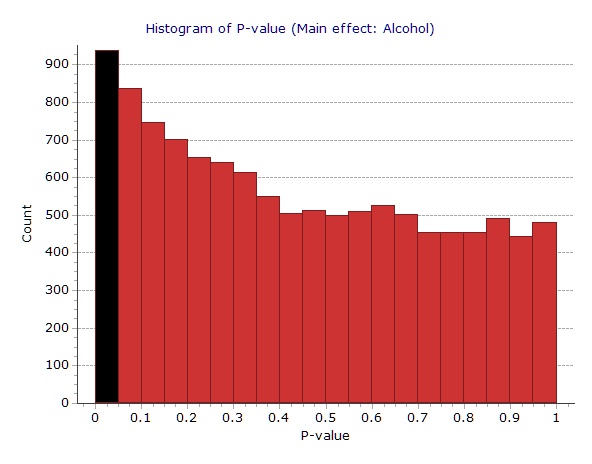


**Supplemental Figure 5:** Histogram of *p-*value distributions for Alcohol divided into 20 bins with each bin representing 0.05 units. The distribution shows a distinct and sharp increase in the 0-0.05 bin indicating that Alcohol as an experimental factor resulted in a higher number of differentially expressed genes than would have been predicted under the null hypothesis. The black shaded bar represents the most frequent bin within the distribution.


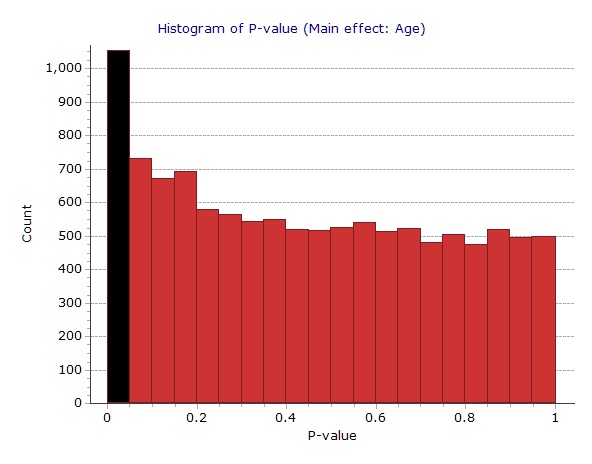


**Supplemental Figure 6:** Histogram of *p-*value distributions using Age as a main effect divided into 20 bins with each bin representing 0.05 units of distribution. The distribution shows a distinct and sharp increase in the 0-0.05 bin indicating that Age as an experimental factor resulted in a higher number of differentially expressed genes than would have been predicted under the null hypothesis. The black shaded bar represents the most frequent bin within the distribution.


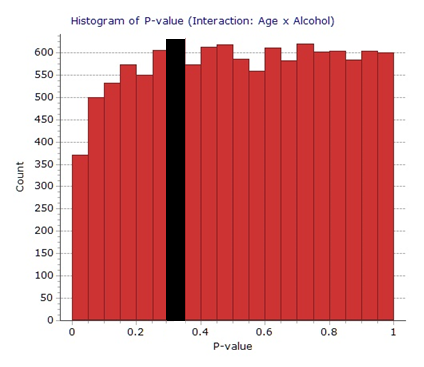


**Supplemental Figure 7:** Histogram of *p-*value distributions for the interaction between Age and Alcohol divided into 20 bins with each bin representing 0.05 units. The distribution shows a flat distribution indicating there is no evidence for a generalized genome wide interaction effect between these two experimental factors.

The black shaded bar represents the most frequent within the distribution.

**
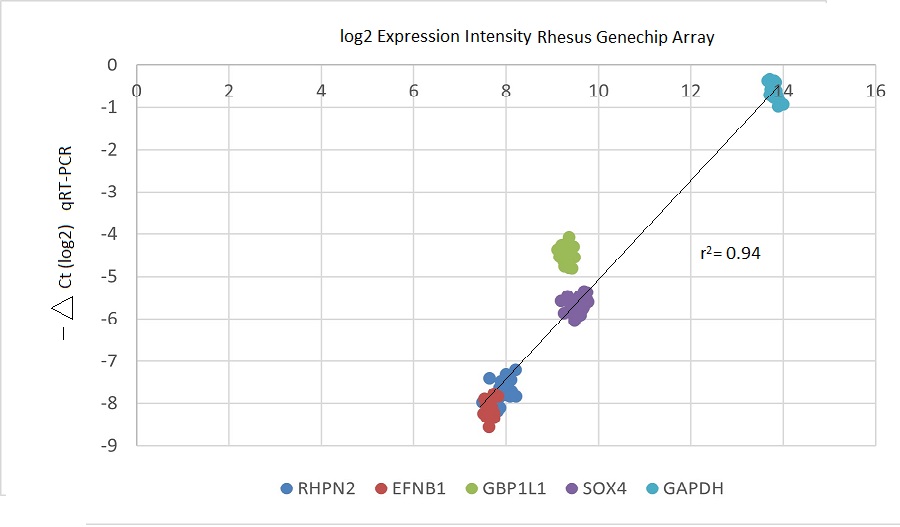
**

**Supplemental Figure 8:** Correlation of log2 expression intensity and -delta Ct values using *ACTB* to normalize expression values. *GBPB1L1* strays from the trend line implying that it may have amplified a target region not represented by the probe set.

**Mean expression (Log2) and mean variance observed across the 4 groups**

|  | Mean expression | Mean variance |
| --- | --- | --- |
| 5 Month Alcohol | 7.95 | 0.04 |
| 5 Month Control | 7.97 | 0.04 |
| 2 Year Alcohol | 7.96 | 0.04 |
| 2 Year Control | 7.96 | 0.04 |

**Supplemental Table 1:** Mean intensity/expression levels (log2) and mean variance for all 11,512 mRNA for all four groups involved in this study.

**Primer sequences used for qRT-PCR Amplification**

| **Gene** | **Forward** | **Reverse** |
| --- | --- | --- |
| *ACTB* | ctggcattgctgacaggat | cagagtacttgcgctcagga |
| *GAPDH* | acgggaagcttgtcatcaat | catcgccccacttgatttt |
| *EFNB1* | tgagcagctgactaccagca | tgtgtggccatcttgacag |
| *GGCT* | tctggccgctccttacag | agttcgccatgtcccactac |
| *GPBP1L1* | ttttgttcctgcttggctaaa | caggtgctctccgtgttttt |
| *RHPN1* | gggtggaactgctgatgac | ggaagaatcgactctcgacaaa |
| *SOX4* | caacgccgagatctccaa | ggatcttgtcgctgtctttga |

**Supplemental Table 2.** Primer sequences used for qRT-PCR amplification of selected genes taken from the Rhesus GeneChip array.

**Eight Functional Annotation Clusters Related to Development**

| **Annotation Cluster 1** | **p-value** | **Benjamini** |
| --- | --- | --- |
| endocrine system development | 0.00057 | 0.51 |
| pancreas development | 0.08180 | 1.00 |
| endocrine pancreas development | 0.12100 | 1.00 |
|  |  |  |
| **Annotation Cluster 2** | **p-value** | **Benjamini** |
| glial cell development | 0.00093 | 0.51 |
| peripheral nervous system development | 0.01840 | 1.00 |
| axon ensheathment | 0.01840 | 1.00 |
| glial cell differentiation | 0.02770 | 1.00 |
| myelination | 0.05630 | 1.00 |
| peripheral nervous system development | 0.37900 | 1.00 |
|  |  |  |
| **Annotation Cluster 3** | **p-value** | **Benjamini** |
| cell migration | 0.00087 | 0.51 |
| movement of cell or subcellular component | 0.00120 | 0.51 |
| localization of cell | 0.00138 | 0.51 |
| cell motility | 0.00138 | 0.51 |
| locomotion | 0.00181 | 0.51 |
| neurogenesis | 0.00601 | 1.00 |
| generation of neurons | 0.00608 | 1.00 |
| cell development | 0.00763 | 1.00 |
| regulation of cell migration | 0.01070 | 1.00 |
| regulation of cell motility | 0.01200 | 1.00 |
| regulation of cellular component movement | 0.01680 | 1.00 |
| neuron differentiation | 0.01850 | 1.00 |
| regulation of cell differentiation | 0.01920 | 1.00 |
| regulation of locomotion | 0.01920 | 1.00 |
| cell projection organization | 0.02760 | 1.00 |
| cell morphogenesis | 0.04680 | 1.00 |
| negative regulation of developmental process | 0.05360 | 1.00 |
| cell morphogenesis involved in differentiation | 0.05970 | 1.00 |
| cellular component morphogenesis | 0.08200 | 1.00 |
| negative regulation of cell migration | 0.08710 | 1.00 |
| negative regulation of locomotion | 0.09200 | 1.00 |
| negative regulation of cellular component movement | 0.09200 | 1.00 |
| regulation of multicellular organismal development | 0.09270 | 1.00 |
| negative regulation of cell motility | 0.10800 | 1.00 |
| blood vessel development | 0.12300 | 1.00 |
| cardiovascular system development | 0.15200 | 1.00 |
| circulatory system development | 0.15200 | 1.00 |
| vasculature development | 0.17700 | 1.00 |
| actin filament bundle assembly | 0.26800 | 1.00 |
| actin filament bundle organization | 0.27900 | 1.00 |
|  |  |  |
| **Annotation Cluster 5** | **p-value** | **Benjamini** |
| cell migration | 0.00087 | 0.51 |
| localization of cell | 0.00138 | 0.51 |
| cell motility | 0.00138 | 0.51 |
| chemotaxis | 0.07690 | 1.00 |
| taxis | 0.07880 | 1.00 |
| receptor binding | 0.25500 | 1.00 |
| positive regulation of cell migration | 0.29000 | 1.00 |
| positive regulation of cell motility | 0.31100 | 1.00 |
| positive regulation of locomotion | 0.33800 | 1.00 |
| positive regulation of cellular component movement | 0.34200 | 1.00 |
|  |  |  |
| **Annotation Cluster 8** | **p-value** | **Benjamini** |
| neuroepithelial cell differentiation | 0.00135 | 0.51 |
| noradrenergic neuron differentiation | 0.00397 | 0.95 |
| sensory organ development | 0.00562 | 1.00 |
| inner ear development | 0.01120 | 1.00 |
| ear development | 0.02370 | 1.00 |
| columnar/cuboidal epithelial cell differentiation | 0.02450 | 1.00 |
| epidermis development | 0.02680 | 1.00 |
| respiratory system development | 0.02990 | 1.00 |
| epithelial cell differentiation | 0.03050 | 1.00 |
| sympathetic nervous system development | 0.03230 | 1.00 |
| epidermal cell differentiation | 0.03530 | 1.00 |
| inner ear morphogenesis | 0.04420 | 1.00 |
| keratinocyte differentiation | 0.04420 | 1.00 |
| sensory organ morphogenesis | 0.05300 | 1.00 |
| camera-type eye morphogenesis | 0.05380 | 1.00 |
| palate development | 0.06530 | 1.00 |
| eye development | 0.06630 | 1.00 |
| camera-type eye development | 0.06630 | 1.00 |
| ear morphogenesis | 0.09080 | 1.00 |
| embryo development | 0.09830 | 1.00 |
| auditory receptor cell differentiation | 0.10400 | 1.00 |
| urogenital system development | 0.11300 | 1.00 |
| eye morphogenesis | 0.11900 | 1.00 |
| lens development in camera-type eye | 0.11900 | 1.00 |
| autonomic nervous system development | 0.12100 | 1.00 |
| skin development | 0.12400 | 1.00 |
| hair cell differentiation | 0.13900 | 1.00 |
| morphogenesis of an epithelium | 0.14900 | 1.00 |
| epithelial cell development | 0.17400 | 1.00 |
| organ morphogenesis | 0.17400 | 1.00 |
| embryonic organ development | 0.21100 | 1.00 |
| tissue morphogenesis | 0.21800 | 1.00 |
| negative regulation of cell proliferation | 0.22400 | 1.00 |
| embryonic organ morphogenesis | 0.24800 | 1.00 |
| columnar/cuboidal epithelial cell development | 0.25300 | 1.00 |
| transcription factor activity, RNA polymerase II core promoter proximal region sequence-specific binding | 0.28500 | 1.00 |
| inner ear receptor cell differentiation | 0.32200 | 1.00 |
| mechanoreceptor differentiation | 0.34100 | 1.00 |
| core promoter binding | 0.35400 | 1.00 |
| focal adhesion | 0.40400 | 1.00 |
| cell-substrate adherens junction | 0.41100 | 1.00 |
| cell-substrate junction | 0.41600 | 1.00 |
| embryonic morphogenesis | 0.44000 | 1.00 |
| RNA polymerase II transcription factor activity, sequence-specific DNA binding | 0.49300 | 1.00 |
| digestive system development | 0.63200 | 1.00 |
|  |  |  |
| **Annotation Cluster 9** | **p-value** | **Benjamini** |
| cell migration | 0.00087 | 0.51 |
| localization of cell | 0.00138 | 0.51 |
| cell motility | 0.00138 | 0.51 |
| regulation of cell migration | 0.01070 | 1.00 |
| regulation of cell motility | 0.01200 | 1.00 |
| regulation of cellular component movement | 0.01680 | 1.00 |
| regulation of locomotion | 0.01920 | 1.00 |
| anatomical structure formation involved in morphogenesis | 0.03150 | 1.00 |
| ephrin receptor signaling pathway | 0.04240 | 1.00 |
| regulation of cell adhesion mediated by integrin | 0.12100 | 1.00 |
| blood vessel development | 0.12300 | 1.00 |
| cardiovascular system development | 0.15200 | 1.00 |
| circulatory system development | 0.15200 | 1.00 |
| angiogenesis | 0.15500 | 1.00 |
| organ morphogenesis | 0.17400 | 1.00 |
| vasculature development | 0.17700 | 1.00 |
| blood vessel morphogenesis | 0.21000 | 1.00 |
| peptidyl-tyrosine phosphorylation | 0.21400 | 1.00 |
| peptidyl-tyrosine modification | 0.22100 | 1.00 |
| positive regulation of kinase activity | 0.22600 | 1.00 |
| regulation of anatomical structure morphogenesis | 0.34800 | 1.00 |
| negative regulation of angiogenesis | 0.36000 | 1.00 |
| negative regulation of blood vessel morphogenesis | 0.37900 | 1.00 |
| negative regulation of vasculature development | 0.40700 | 1.00 |
| regulation of angiogenesis | 0.48600 | 1.00 |
| transmembrane receptor protein tyrosine kinase signaling pathway | 0.53800 | 1.00 |
| negative regulation of intracellular signal transduction | 0.54800 | 1.00 |
| endothelial cell differentiation | 0.56700 | 1.00 |
| regulation of vasculature development | 0.57400 | 1.00 |
| vascular endothelial growth factor receptor signaling pathway | 0.57500 | 1.00 |
| endothelium development | 0.64500 | 1.00 |
|  |  |  |
| **Annotation Cluster 13** | **p-value** | **Benjamini** |
| respiratory system development | 0.02990 | 1.00 |
| epithelial cell differentiation | 0.03050 | 1.00 |
| lung epithelium development | 0.03950 | 1.00 |
| lung epithelial cell differentiation | 0.07200 | 1.00 |
| lung cell differentiation | 0.07970 | 1.00 |
| lung development | 0.10200 | 1.00 |
| respiratory tube development | 0.11100 | 1.00 |
| transcription initiation from RNA polymerase II promoter | 0.44600 | 1.00 |
| RNA polymerase II transcription factor activity, sequence-specific DNA binding | 0.49300 | 1.00 |
| DNA-templated transcription, initiation | 0.62400 | 1.00 |
|  |  |  |
| **Annotation Cluster 15** | **p-value** | **Benjamini** |
| gliogenesis | 0.00692 | 1.00 |
| central nervous system development | 0.01410 | 1.00 |
| limbic system development | 0.06140 | 1.00 |
| cerebral cortex cell migration | 0.06760 | 1.00 |
| telencephalon cell migration | 0.12500 | 1.00 |
| cerebral cortex radially oriented cell migration | 0.13000 | 1.00 |
| forebrain cell migration | 0.14300 | 1.00 |
| forebrain development | 0.15600 | 1.00 |
| cerebral cortex development | 0.17300 | 1.00 |
| telencephalon development | 0.18000 | 1.00 |
| pallium development | 0.21700 | 1.00 |
| brain development | 0.24800 | 1.00 |
| transcription factor activity, RNA polymerase II core promoter proximal region sequence-specific binding | 0.28500 | 1.00 |
| head development | 0.32000 | 1.00 |
| hippocampus development | 0.47800 | 1.00 |

**Supplementary Table 3:** Functional annotation results using the 297 genes which returned the lowest *p*-values using Age as a main effect. Eight of the 15 top annotation clusters returned results related to development or cell migration with lower overall *p*-values and FDR values when compared to those generated using alcohol as a main effect.
